# Optimized synthesis of aroyl-*S,N*-ketene acetals by omission of solubilizing alcohol cosolvents

**DOI:** 10.3762/bjoc.21.97

**Published:** 2025-06-20

**Authors:** Julius Krenzer, Thomas J J Müller

**Affiliations:** 1 Heinrich-Heine-Universität Düsseldorf, Institut für Organische Chemie und Makromolekulare Chemie, Universitätsstraße 1, D-40225 Düsseldorf, Germany

**Keywords:** aroyl chlorides, aroyl-*S*,*N*-ketene acetals, condensation, Einhorn-type acylation, 2-methyl *N*-benzyl benzothiazolium salts

## Abstract

Aroyl-*S*,*N*-ketene acetals are obtained by condensation of aroyl chlorides and 2-methyl-*N*-benzylbenzothiazolium salts in 1,4-dioxane at room temperature in short reaction time in 20–99% yield. This protocol represents a considerable improvement over the standard synthesis in 1,4-dioxane/ethanol mixtures at elevated temperatures.

## Introduction

**Scheme 1:**
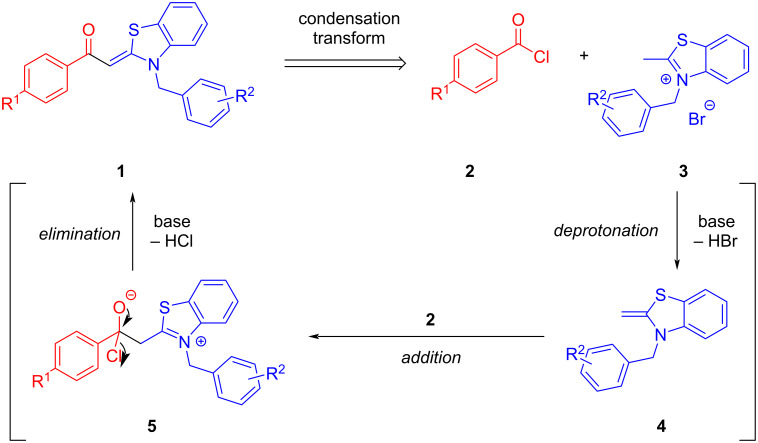
Retrosynthetic analysis of aroyl-*S*,*N*-ketene acetals **1** and tentative mechanistic scenario of the addition–elimination sequence.

Aroyl-*S*,*N*-ketene acetals, in particular *N*-benzyl derivatives **1**, are very short donor–acceptor chromophores that have recently found a renaissance due to their peculiar intense solid-state emission and significant turn-on of emission upon induced aggregation in alcohol–water mixtures [1]. This chromophore class has been extensively developed in recent years, even to aggregation-induced emissive (AIE) multichromophores [2] and even bichromophoric fluorimetric sensors [3–4]. The retrosynthesis of the title compounds **1** suggests starting from aroyl chlorides **2** and 2-methyl-*N*-benzylbenzothiazolium salts **3** by a condensation transform (Scheme 1). The condensation essentially represents an addition–elimination sequence that starts with the nucleophilic *S*,*N*-ketene acetal **4**, in situ generated from substrate **3** by deprotonation, followed by chloride elimination/neutralization from the zwitterionic tetrahedral intermediate **5** to give the target molecule **1**.

**Scheme 2:**
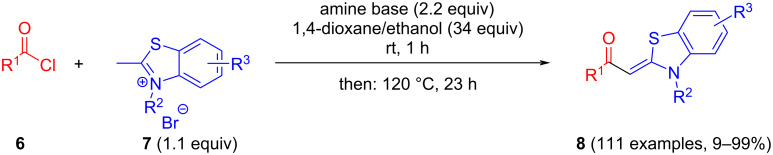
Standard protocol for the synthesis of (hetero)aroyl-*S*,*N*-ketene acetals **8** in binary dioxane/ethanol mixtures.

The standard protocol for the synthesis of (hetero)aroyl-*S*,*N*-ketene acetals **8** from (hetero)aroyl chlorides **6** and 2-methylbenzothiazolium salts **7** employs a twofold excess of an amine base in a binary 1,4-dioxane/ethanol mixture. Ethanol was used as a cosolvent to ensure solubility of the polar intermediate according to the mechanistic rationale (Scheme 2) [5–6]. Although, a broad scope of diversely substituted (hetero)aroyl-*S*,*N*-ketene acetals **8** was obtained (111 examples), the average yield of 57% indicates that the process might require optimization, in particular, for further methodological implementation. Here, we report on the improved synthesis of (hetero)aroyl-*S*,*N*-ketene acetals **8** by careful solvent and temperature optimization.

## Results and Discussion

The presumed byproducts in the addition–elimination sequence in the presence of an excess of ethanol as a cosolvent are the ethyl ester formed by Einhorn acylation [7] of the acid chloride under the standard conditions and deep colored polar byproducts (according to TLC detection) that arise from self-condensation of 2-methylbenzothiazolium salts **7** and intermediary formed *S*,*N*-ketene acetals **4** at elevated temperatures. Einhorn acylation is governed by the electrophilicity of the acid chloride or acylammonium species and the nucleophilicity of any nucleophilic species present in the reaction mixture. In addition, since condensations are affected by the bimolecular addition as an elementary step, special attention to the concentration of the nucleophiles has to be paid.

According to Mayr’s nucleophilicity scales [8–10], a nucleophilicity parameter *N* for the specific *S*,*N*-ketene acetal intermediate **4** can estimated from parameters for enamines (*N* = 10–16), cyclic *N*,*N*-ketene acetals (*N* = 18–20) [11–12] , and the deoxy-Breslow intermediate (*N* =15.6) [13]. The nucleophilicity of *S*,*N*-ketene acetal **4** clearly exceeds that of ethanol (*N* = 7.4). In addition, triethylamine is more nucleophilic (*N* = 17.30 in dichloromethane; *N* = 17.10 in acetonitrile) [14] than ethanol or the *S*,*N*-ketene acetal intermediate **4**. Hence, mechanistically the acid chloride first transforms to an acylammonium species as in Einhorn acylations [7]. As a consequence, for avoiding any competing ethyl ester formation, ethanol has to be omitted from in the process. For suppressing the formation of side products by self-condensation of *S*,*N*-ketene acetal intermediates the reaction temperature has to be kept as low as possible for assuring kinetic control.

**Scheme 3:**
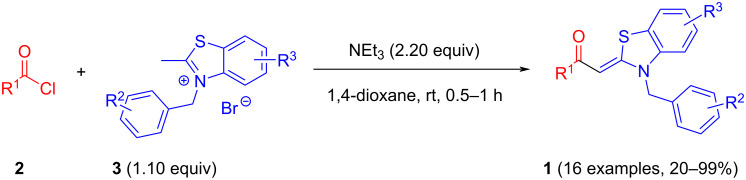
Modified protocol for the synthesis of aroyl-*S*,*N*-ketene acetals **1** in dioxane at room temperature.

Therefore, in contrast to the standard protocol [5–6] (Scheme 2), reacting aroyl chlorides **2** and 2-methyl-*N*-benzylbenzothiazolium salts **3** in 1,4-dioxane as a solvent at room temp for 0.5 to 1 h produces representative aroyl-*S*,*N*-ketene acetals **1** in mostly excellent yield (Scheme 3). For comparison, the yields of the products **1** under standard (A) and new modified (B) conditions are listed in Table 1. The yield of compounds **1** after flash chromatography on silica gel under modified conditions (B) are significantly higher than under standard conditions (A).

**Table 1:** Comparison of the yields of the condensation synthesis of aroyl-*S*,*N*-ketene acetals **1** under standard (A)^a^ and modified conditions B^b^ and C^c^.

entry	acid chloride **2**	benzothiazolium salt **3**	aroyl-*S*,*N*-ketene acetal **1**^d^

**1**	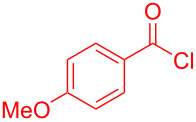 **2a**	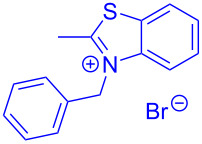 **3a**	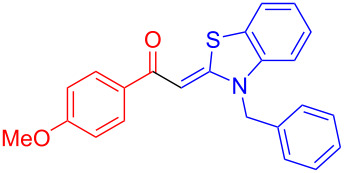 **1a** (A: 52% [5]; B: 90%)
**2**	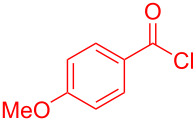 **2a**	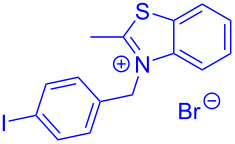 **3b**	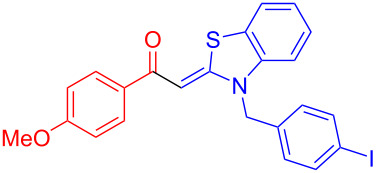 **1b** (B: 68%)
**3**	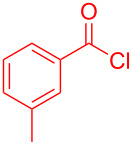 **2b**	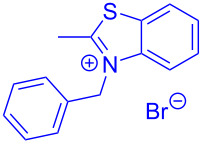 **3a**	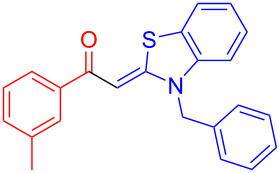 **1c** (A: 45% [6]; B: 96%)
**4**	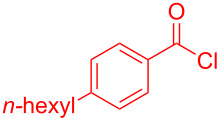 **2c**	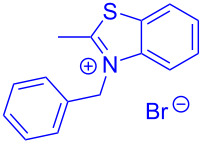 **3a**	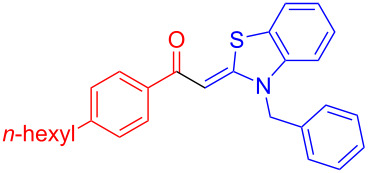 **1d** (A: 44% [6]; B: 95%)
**5**	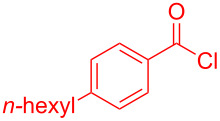 **2c**	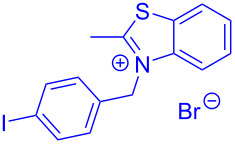 **3b**	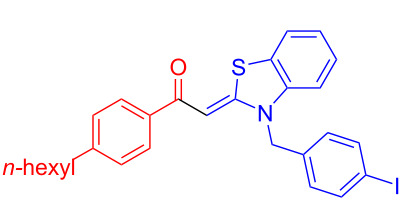 **1e** (A: 51%; B: 81%)
**6**	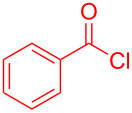 **2d**	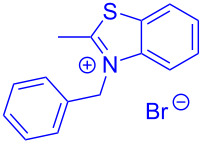 **3a**	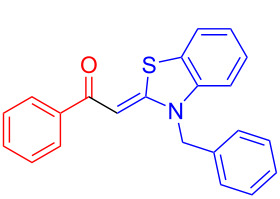 **1f** (A: 58% [5]; B: 93%; C: 95%)
**7**	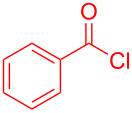 **2d**	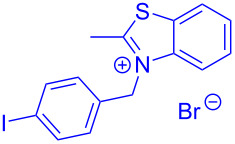 **3b**	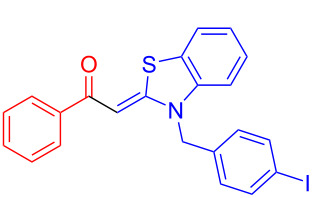 **1g** (A: 61% [6]; B: 99%)
**8** ^e^	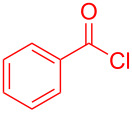 **2d**	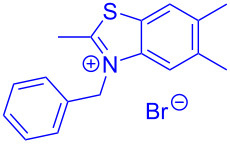 **3d**	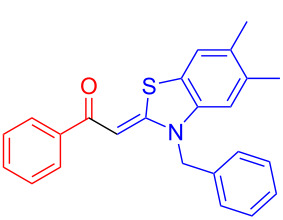 **1h** (A: 75% [6]; B: 92%; C: 91%)
**9**	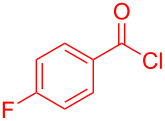 **2e**	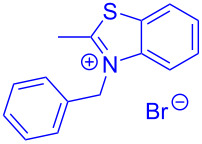 **3a**	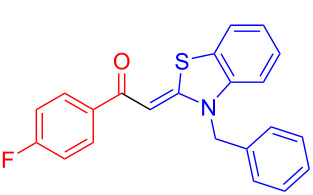 **1i** (A: 85% [5]; B: 99%)
**10**	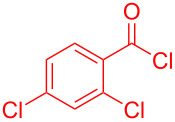 **2f**	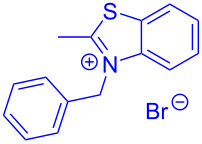 **3a**	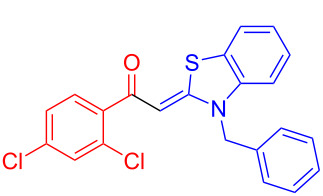 **1j** (A: 38% [6]; B: 98%)
**11**	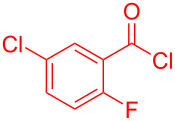 **2g**	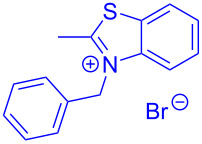 **3a**	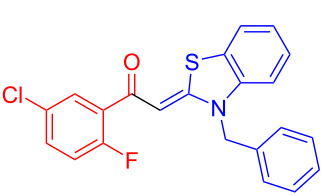 **1k** (A: 27% [6]; B: 99%)
**12**	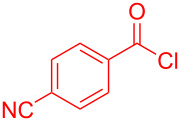 **2h**	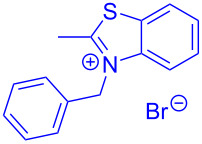 **3a**	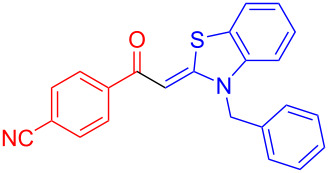 **1l** (A: 66% [5]; B: 97%)
**13**	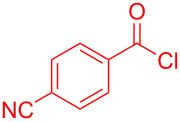 **2h**	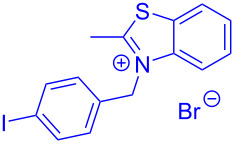 **3b**	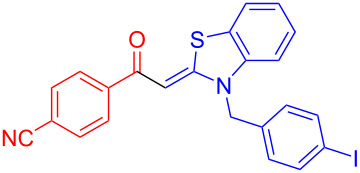 **1m** (A: 32% [6]; B: 53%)
**14**	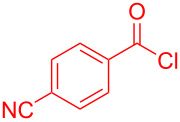 **2h**	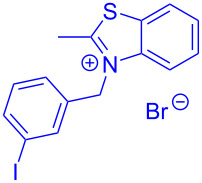 **3c**	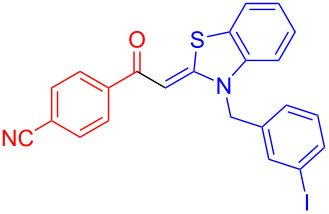 **1n** (A: 45%; B: 87%)
**15**	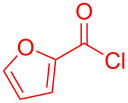 **2i**	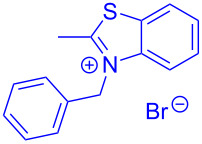 **3a**	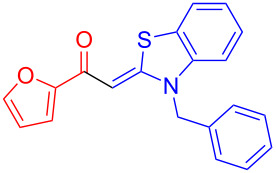 **1o** (A: 95% [5]; B: 98%)
**16**	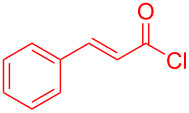 **2j**	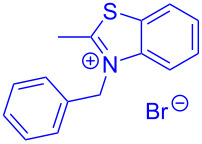 **3a**	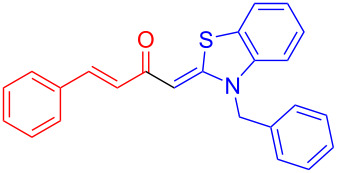 **1p** (A: 11% [6]; B: 20%)

^a^Conditions A: Aroyl chloride **2** (1.0 equiv), 2-methyl-*N*-benzylbenzothiazolium salts **3** (1.1 equiv), and triethylamine (2.2 equiv) in a mixture of 1,4-dioxane and ethanol (34 equiv) were stirred at room temperature for 1 h and then at 120 °C for 23 h. ^b^Conditions B: Aroyl chloride **2** (1.0 equiv), 2-methyl-*N*-benzylbenzothiazolium salts **3** (1.1 equiv), and triethylamine (2.2 equiv) in 1,4-dioxane were stirred at room temperature for 0.5 to 1 h. ^c^Conditions C: Aroyl chloride **2** (1.0 equiv), 2-methyl-*N*-benzylbenzothiazolium salts **3** (1.1 equiv), and triethylamine (2.2 equiv) in 2-Me-THF were stirred at room temperature for 1 h. ^d^Yields after chromatography on silica gel. ^e^A dilution to 8 mL of 1,4-dioxane per mmol was used.

The results demonstrate the broad applicability of the optimized reaction conditions. Acid chlorides with electron-withdrawing (-CN) and electron-donating (-OMe) substituents were used. Additionally, a heterocycle could be incorporated. Different benzyl substituents were employed, and substitution at the benzothiazole core was also tolerated (Table 1).

As an alternative to 1,4-dioxane, 2-methyltetrahydrofuran was tested the solvent. This can be obtained from renewable biomass and is therefore considered to be a "green" solvent. In 2-MeTHF (conditions (C)), product **1f** was isolated with a yield of 95% and derivative **1h** with a yield of 91%. This shows that the sustainable solvent is a potent alternative to 1,4-dioxane. With the new protocol, not only does the synthesis need less energy, it can also be carried out more sustainably, making it "greener."

## Conclusion

Changing the solvent system from 1,4-dioxane/ethanol to 1,4-dioxane and reacting the aroyl chlorides and 2-methyl-*N*-benzylbenzothiazolium salts at room temp for 0.5–1 h gives rise to the formation of aroyl-*S*,*N*-ketene acetals in mostly excellent yield. The modification of this condensation synthesis is conceptualized on the basis of relative reactivities of intermediary formed acylammonium salts (electrophilicity) and *S*,*N*-ketene acetals (nucleophilicity). In addition, kinetic control is warranted by conducting the condensation synthesis at room temperature for only short reaction times. Furthermore, 2-MeTHF was successfully implemented as a sustainable alternative to 1,4-dioxane. This modified protocol is clearly superior to the initial protocol (in 1,4-dioxane/ethanol) and will be applied in future sequences for the generation of (hetero)aroyl-*S*,*N*-ketene acetals.

## Experimental

**Synthesis of compound 1i according to conditions B (typical procedure):** 4-Fluorobenzoyl chloride (**2e**, 0.590 mL, 4.99 mmol, 1.00 equiv) and benzothiazolium salt **3a** (1.76 g, 5.50 mmol, 1.10 equiv) were placed in a sintered, dry screw-cap Schlenk-tube under nitrogen atmosphere and dissolved in dry 1,4-dioxane (30 mL). Triethylamine (1.52 mL, 11.0 mmol, 2.20 equiv) was added to the reaction mixture and the solution was stirred for 1 h at room temperature. The crude product was absorbed onto Celite^®^ and purified by flash chromatography on silica gel (*n*-hexane/acetone 3:1). Then, the crude product was suspended in *n*-hexane, the supernatant separated by filtration and the precipitate was dried under vacuum to afford aroyl-*S*,*N*-ketene acetal **1i** (1.78 g, 4.93 mmol, 99%) as a yellow solid. Mp 159 °C (lit. 155 °C [5]). ^1^H NMR (300 MHz, CDCl_3_) δ 5.24 (s, 2H), 6.43 (s, 1H), 6.92–7.04 (m, 3H), 7.09–7.16 (m, 3H), 7.19–7.29 (m, 4H), 7.57 (dd, ^3^*J* = 7.7 Hz, ^4^*J* = 1.3 Hz, 1H), 7.73–7.83 (m, 2H); MALDI–TOF–MS (*m/z*): 362.2, [C_22_H_16_FNOS + H]^+^.

## Supporting Information

File 1 Experimental details of the synthesis and analytical data of compounds **1** and **3**, ^1^H and ^13^C NMR spectra of compounds **1** and **3**.

## Data Availability

All data that supports the findings of this study is available in the published article and/or the supporting information of this article.
