# Supplementary material for: Optimized synthesis of aroyl-S,N-ketene acetals by omission of solubilizing alcohol cosolvents
Source: Beilstein J Org Chem. 2025 Jun 20;21:1201–6. doi: 10.3762/bjoc.21.97 (PMC12207251; doi:10.3762/bjoc.21.97)
Supplement: File 1 — Experimental details of the synthesis and analytical data of compounds 1 and 3, 1H and 13C NMR spectra of compounds 1 and 3. [file Beilstein_J_Org_Chem-21-1201-s001.pdf]

BEILSTEIN JOURNAL OF ORGANIC CHEMISTRY

Supporting Information

for

Optimized synthesis of aroyl-*S,N*-ketene acetals by omission of solubilizing alcohol cosolvents

Julius Krenzer and Thomas J. J. Müller

Beilstein J. Org. Chem. **2025,** *21,* 1201–1206. doi:10.3762/bjoc.21.97

Experimental details of the synthesis and analytical data of compounds 1 and 3, ^{1}H and ^{13}C NMR spectra of compounds 1 and 3

License and Terms: This is a supporting information file under the terms of the Creative Commons Attribution License (https://creativecommons.org/licenses/by/4.0). Please note that the reuse, redistribution and reproduction in particular requires that the author(s) and source are credited and that individual graphics may be subject to special legal provisions.
The license is subject to the *Beilstein Journal of Organic Chemistry* terms and conditions: (https://www.beilstein-journals.org/bjoc/terms)

Table of contents

1 General considerations S1

2 Syntheses S3

2.1 Synthesis of benzothiazolium bromides 3 .... S3

2.1.1 General procedure (GP I) S3

2.1.2 Spectroscopic data S4

2.2 Synthesis of aroyl-*S*,*N*-ketene acetals 1 .... S7

2.2.1 General procedure (GP II) S7

2.2.2 Spectroscopic data S10

3 NMR spectra S20

3.1 NMR spectra of benzothiazolium bromide 3c S20

3.2 NMR spectra of aroyl-*S*,*N*-ketene acetals 1 .... S21

4 Literature S24

1 General considerations

Reactions were carried out in dried and sintered Schlenk tubes or round bottom flasks under nitrogen atmosphere. Solvents were dried by a solvent purification system *MB-SPS-800* from *MBraun Inertgas-Systeme GmbH*.

The used chemicals which have not been synthesized were purchased at *Acros Organics BVBA*, *Alfa Aeser GmbH & Co KG*, *Fluorochem Ltd.*, *J&K Scientific Ltd.*, *Merck KGaA*, *Macherey-Nagel GmbH & Co. KG*, *Sigma-Aldrich Chemie GmbH* and *VWR* and have been used without further purification.

Further purification of the compounds was performed by flash column chromatography (silica gel M60 pore size 0.040–0.063 nm) from *Macherey-Nagel*. The crude product was adsorbed on Celite®545 from *Carl Roth GmbH*, placed on the suspended silica gel and purified with a positive pressure of 1 bar. Distilled solvent mixtures of *n*-hexane and acetone have been used as eluents.

The control of reaction progress was done via thin layer chromatography (TLC) with silica coated aluminium plates F_{254}, from *Macherey-Nagel GmbH & Co. KG.*

The melting points have been measured with a *Melting Point B-540* apparatus from *Büchi* according to the protocol of *Kofler*. [1]

^{1}H,^{13}C and DEPT 135-spectra have been measured at 298 K on an *Avance III - 300* and an *Avance III - 600* from *Bruker*. Chemical shifts in the ^{1}H and ^{13}C NMR are reported in ppm relative to deuterated solvents such as chloroform-d_1 (δ_H 7.26, δ_C 77.23) and DMSO-d_6 (δ_H 2.50, δ_C 39.51). [2] The multiplicity is abbreviated as followed: s = singlet; d = doublet; t = triplet; td = triplet of doublet; dd = doublet of doublet; dt = doublet of triplet, m = multiplet. The assignment of primary carbon centers (CH), secondary carbon centers (CH_2), tertiary carbon centers (CH_3) and quaternary carbon centers (C_{quat}) were made by using DEPT-135 spectra.

All mass spectrometry experiments have been performed by the department for mass spectrometry of the University of Düsseldorf (HHUCeMSA). EI mass spectra have been measured with the Triple-Quadrupol-spectrometer *TSQ 7000* from *Finnigan MAT*. MALDI spectra have been measured with a *MALDI/TOF UltrafleXtreme* from *Bruker Daltronik*.

IR spectra were recorded with neat compounds under attenuated total reflection (ATR) with *IRAffinity-1* from *Shimadzu* and the intensities were characterized as strong (s), middle (m) and weak (w).

The elementary analyses have been measured with *Perkin Elmer Series II Analyser 2400* or *Vario Micro Cube* from *Analysensysteme GmbH* at the microanalytical laboratory of the institute for Pharmaceutical and Medicinal Chemistry of the University Düsseldorf.

2 Syntheses

2.1 Synthesis of benzothiazolium bromides 3

2.1.1 General procedure (GP I)

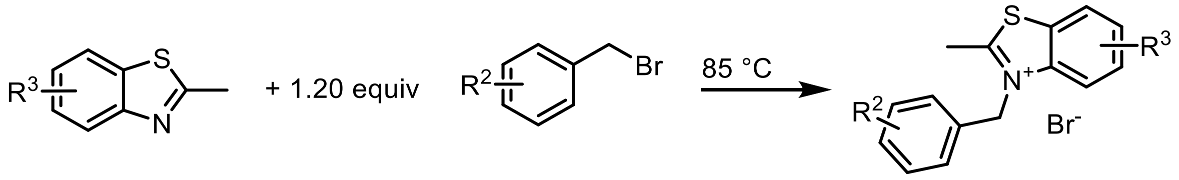

3

In a protocol analogous to Yen [3], 2-methylbenzothiazole (1.00 equiv) and a benzyl bromide (1.20 equiv) were placed in a round-bottom flask (for experimental details, see Table S1). The reaction mixture was stirred at 85 °C for 24 h, until the solution was completely solidified. The formed solid was filtrated via a Buechner funnel, washed with diethyl ether and dried under vacuo.

Table S1: Experimental details for the synthesis of benzothiazolium bromides **3**.

entry	2-methylbenzothiazole	benzylbromide	benzothiazolium bromide **3** (Yield)
**1**	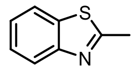<br>1.27 mL<br>(9.98 mmol)	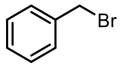<br>1.43 mL<br>(12.00 mmol)	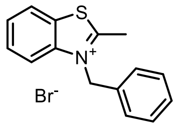<br>2.49 g (78%) of **3a**
**2**	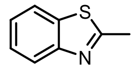<br>1.27 ml<br>(9.98 mmol)	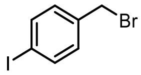<br>3.56 g<br>(12.0 mmol)	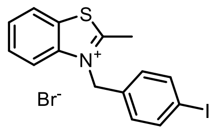<br>4.34 g (98%) of **3b**
**3**	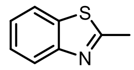<br>1.13 ml<br>(8.88 mmol)	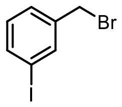<br>3.19 g<br>(10.7 mmol)	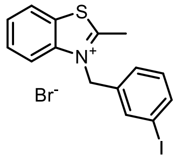<br>2.71 g (68%) of **3c**
**4**	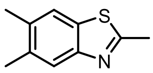<br>1.77 g<br>(9.99 mmol)	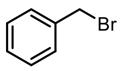<br>1.43 ml<br>(12.00 mmol)	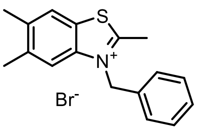<br>2.96 g (85%) of **3d**

2.1.2 Spectroscopic data

3-Benzyl-2-methylbenzo[*d*]thiazol-3-ium bromide (3a) [3-4]

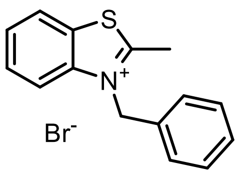

$C_{15}H_{14}BrNS$ [320.25]

The synthesis was performed according to **GP I** to give 2.49 g (7.77 mmol, 78%) of the desired product as a colorless solid, Mp 234 °C (lit. 200 °C [4]). R_f (*n*-hexane/acetone 1:1): 0.10.

^{1}H NMR (300 MHz, DMSO-d$_6$): δ 3.27 (s, 3 H), 6.10 (s, 2 H), 7.23 - 7.51 (m, 5 H), 7.71 - 7.95 (m, 2 H), 8.20 (d, ^3J = 7.1 Hz, 1 H), 8.51 (d, ^3J = 7.6 Hz, 1 H).

^{13}C NMR (75 MHz, DMSO-d$_6$): δ 17.3 (CH_3), 51.9 (CH_2), 117.1 (CH), 124.9 (CH), 127.0 (CH), 128.2 (C_{quat}), 128.6 (CH), 129.1(CH), 129.3 (C_{quat}), 129.5(CH), 132.8 (C_{quat}), 141.0, 178.4 (C_{quat}).

EI MS (70 eV, *m*/*z* (%)): 240 ($[C_{15}H_{14}NS]^+$, 20), 239 ($[C_{15}H_{13}NS]^+$, 92), 238 ($[C_{15}H_{12}NS]^+$, 99), 224 (18), 162 (17), 148 ($[C_8H_6NS]^+$, 37), 104 (14), 91 ($[C_7H_7]^+$, 100), 65 (21).

3-(4-Iodobenzyl)-2-methylbenzo[*d*]thiazol-3-ium bromide (3b) [5]

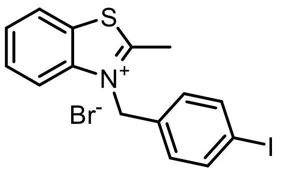

$C_{15}H_{13}BrINS$ [446.14]

The synthesis was performed according to **GP I** to give 4.34 g (9.74 mmol, 98%) of the desired product as a colorless solid, Mp 229 °C (lit. 211 °C [5]), R_f (*n*-hexane/acetone 1:1): 0.10.

^{1}H NMR (300 MHz, DMSO-d$_6$): δ 3.24 (s, 3 H), 6.05 (s, 2 H), 7.14 (d, ^3J = 8.4 Hz, 2 H), 7.72 - 7.88 (m, 4 H), 8.17 (d, ^3J = 7.8, 1 H), 8.50 (d, ^3J = 7.7 Hz, 1 H).

^{13}**C NMR (75 MHz, DMSO-d$_6$):** δ 17.3 (CH_3), 51.4 (CH_2), 95.0 (C_{quat}), 117.0 (CH), 124.9 (CH), 128.2 (CH), 129.3 (C_{quat}), 129.3 (CH), 129.6 (CH), 132.6 (C_{quat}), 137.8 (CH), 140.9 (C_{quat}), 178.7 (C_{quat}).

EI MS (70 eV, *m*/*z* (%)): 366 ($[C_{15}H_{13}INS]^+$, 14), 365 ($[C_{15}H_{13}NS]^+$, 81), 364 ($[C_{15}H_{12}NS]^+$, 49), 238 ($[C_{15}H_{12}NS]^+$, 14), 223 (11), 217 ($[C_7H_6I]^+$, 100), 162 (11), 148 ($[C_8H_6NS]^+$, 32), 119 (12), 118 (18), 104 (11), 90 ($[C_7H_7]^+$, 34), 89 ($[C_7H_6]^+$, 29).

3-(3-Iodobenzyl)-2-methylbenzo[*d*]thiazol-3-ium bromide (3c)

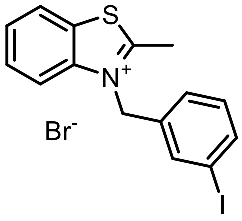

$C_{15}H_{13}BrINS$ [446.14]

The synthesis was performed according to **GP I** to give 2.71 g (6.08 mmol, 68%) of the desired product as a colorless solid, Mp 233 °C. R_f (*n*-hexane/acetone 1:1): 0.10.

^{1}**H NMR (300 MHz, DMSO-d$_6$):** δ 3.25 (s, 3 H), 6.06 (s, 2 H), 7.12 - 7.26 (m, 2 H), 7.71 - 7.89 (m, 4 H), 8.16 (d, ^3J = 7.6 Hz, 1 H), 8.51 (d, ^3J = 7.8 Hz, 1 H).

^{13}**C NMR (75 MHz, DMSO-d$_6$):** δ 17.4 (CH_3), 51.0 (CH_2), 95.6 (C_{quat}), 116.9 (CH), 124.9 (CH), 126.1(CH), 128.2 (CH) , 129.3 (C_{quat}), 129.6 (CH), 131.1 (CH) , 135.2 (C_{quat}), 135.6 (CH), 137.3 (CH), 140.9 (C_{quat}), 178.9 (C_{quat}).

EI MS (70 eV, *m*/*z* (%)): 366 ($[C_{15}H_{13}INS]^+$, 20), 365 ($[C_{15}H_{12}INS]^+$, 95), 364 ($[C_{15}H_{11}INS]^+$, 100), 350 (19), 239 ($[C_{15}H_{13}NS]^+$, 12), 238 ($[C_{15}H_{12}NS]^+$, 61), 237 ($[C_{15}H_{11}NS]^+$, 17), 236 (21), 223 (34), 217 ($[C_7H_6I]^+$, 38), 162 (20), 149 ($[C_8H_7NS]^+$, 23), 148 ($[C_8H_6NS]^+$, 89), 119 (21), 118 (32), 112 (16), 108 (10), 104 (22), 90 ($[C_7H_7]^+$, 37), 89 ($[C_7H_6]^+$, 41), 82 (15), 80 (10), 77 (11), 63 (12), 45 (14), 44 (20).

IR $\tilde{\nu}$ [cm^{-1}]: 3009 (w), 2949 (w), 2891 (w), 2864 (w), 2743 (w), 1591 (w), 1566 (m), 1510 (w), 1476 (m), 1441 (m), 1420 (m), 1368 (w), 1304 (w), 1277 (w), 1244 (w), 1204 (m), 1165 (w), 1096 (w), 1063 (m), 943 (w), 899 (m), 837 (w), 808 (m), 771 (s), 733 (s), 716 (m), 704 (s), 683 (m), 656 (s).

Anal calcd for $C_{15}H_{13}BrINS$ [446.1]: C 40.38, H 2.94, N 3.14, S 7.19; **Found:** C 40.61, H 3.00, N 3.14, S 7.39.

3-Benzyl-2,5,6-trimethylbenzo[*d*]thiazol-3-ium bromide (3d) [5]

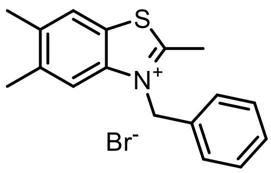

$C_{17}H_{18}BrNS$ [348.30]

The synthesis was performed according to **GP I** to give 2.96 g (8.45 mmol, 85%) of the desired product as a colorless solid, Mp 225 °C (lit. 240 °C) [5]. R_f (*n*-hexane/acetone 1:1): 0.10.

^{1}H NMR (600 MHz, DMSO-d$_6$): δ 2.40 (s, 3 H), 2.41 (s, 3 H), 3.19 (s, 3 H), 6.03 (s, 2 H), 7.25 - 7.30 (m, 2 H), 7.33 - 7.43 (m, 3 H), 8.07 (s, 1 H), 8.21 (s, 1 H).

^{13}C NMR (150 MHz, DMSO-d$_6$): δ 17.0 (CH_3), 19.7 (CH_3), 20.0 (CH_3), 51.7 (CH_2), 116.7 (CH), 123.9 (CH), 126.6 (C_{quat}), 126.9 (CH), 128.5 (CH), 129.1 (CH), 132.8 (C_{quat}), 138.2 (C_{quat}), 139.6 (C_{quat}), 139.6 (C_{quat}), 176.4 (C_{quat}).

EI MS (70 eV, *m/z* (%)): 268 ($[C_{17}H_{18}NS]^+$, 29), 267 ($[C_{17}H_{16}NS]^+$, 100), 266 ($[C_{17}H_{18}NS]^+$, 47), 252 (14), 178 ($[C_{10}H_{12}NS]^+$, 11), 177 ($[C_{10}H_{11}NS]^+$, 39), 176 ($[C_{10}H_{10}NS]^+$, 68), 162 (13), 132 (16), 92 ($[C_7H_8]^+$, 11), 91 ($[C_7H_7]^+$, 84), 65 (22).

2.2 Synthesis of aroyl-*S*,*N*-ketene acetals 1

2.2.1 General procedure (GP II)

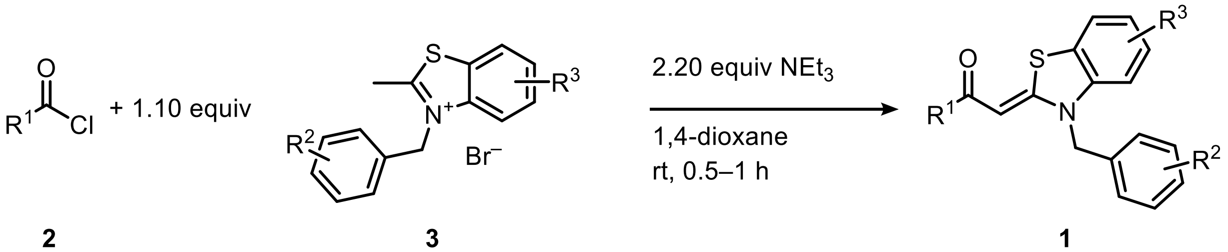

2 **3** **1**

Acid chloride **2** (1.00 equiv) and benzothiazolium salt **3** (1.10 equivs) were placed in a sintered, dry screw-cap Schlenk-tube with magnetic stir bar under nitrogen atmosphere and dissolved in dry 1,4-dioxane (6 mL/mmol) (for experimental details, see Table S2). 2.20 equiv amine base was added to the reaction mixture and the solution was stirred for 0.5–1 h at room temperature. The crude product was absorbed onto Celite® and purified by flash chromatography on silica gel (*n*-hexane/acetone 3:1). The product was suspended in *n*-hexane, the supernatant separated by filtration and the precipitate was dried dried under vacuo.

Table 2S. Experimental details for the synthesis of aroyl-*S*,*N*-ketene acetals

entry	acid chloride **2**	benzothiazolium bromide **3**	**1** (yield)
1	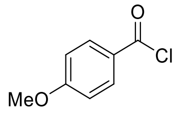 853 mg (5.00 mmol) of **2a**	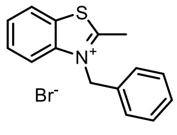 1.76 g (5.50 mmol) of **3a**	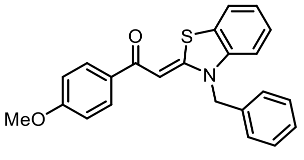 1.68 g (90%) of **1a**
2	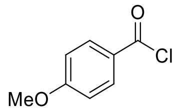 853 mg (5.00 mmol) of **2a**	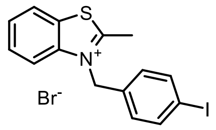 2.45 g (5.49 mmol) of **3b**	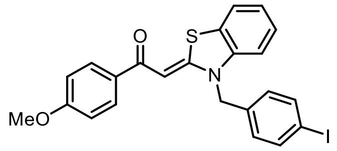 1.71 g (68%) of **1b**
3	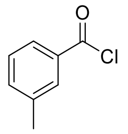 0.130 mL (0.984 mmol) of **2b**	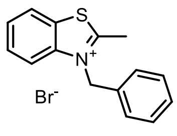 352 mg (1.10 mmol) of **3a**	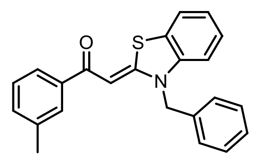 339 mg (96%) of **1c**
4	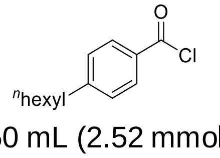 0.550 mL (2.52 mmol) of **2c**	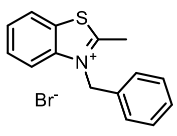 887 mg (2.77 mmol) of **3a**	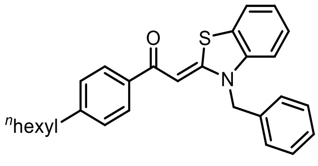 1.02 g (95%) of **1d**

entry	acid chloride **2**	benzothiazolium bromide **3**	**1** (yield)
5	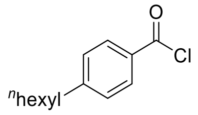 1.09 mL (4.99 mmol) of **2c**	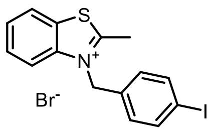 2.45 g (5.49 mmol) of **3b**	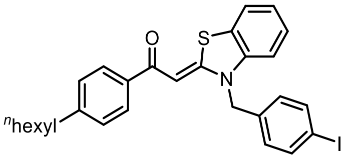 2.24 g (81%) of **1e**
6	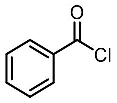 0.120 mL (1.03 mmol) of **2d**	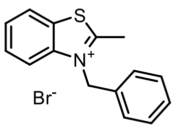 352 mg (1.10 mmol) of **3a**	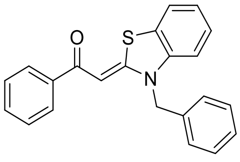 329 mg (93%) of **1f**
7	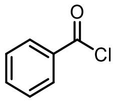 0.580 mL (4.99 mmol) of **2d**	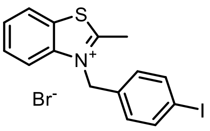 2.45 g (5.49 mmol) of **3b**	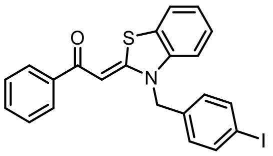 2.32 g (99%) of **1g**
8[a]	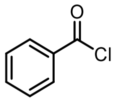 0.120 mL (1.03 mmol) of **2d**	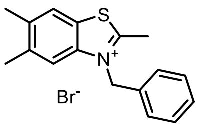 385 mg (1.10 mmol) of **3d**	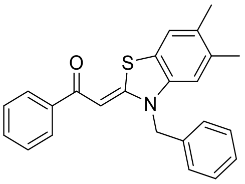 353 mg (92%) of **1h**,
9	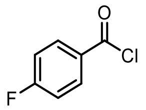 0.590 mL (4.99 mmol) of **2e**	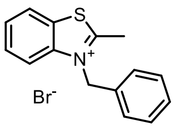 1.76 g (5.50 mmol) of **3a**	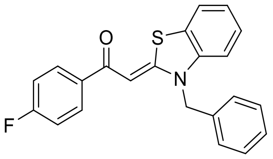 1.78 g (99%) of **1i**
10	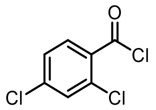 0.140 mL (0.996 mmol) of **2f**	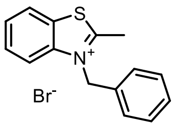 352 mg (1.10 mmol) of **3a**	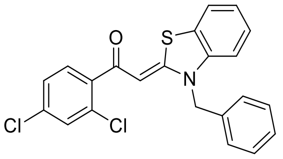 403 mg (98%) of **1j**
11	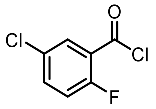 0.130 mL (0.983 mmol) of **2g**	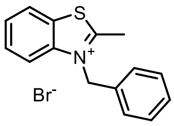 352 mg (1.10 mmol) of **3a**	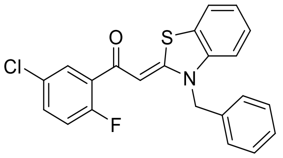 387 mg (99%) of **1k**
12	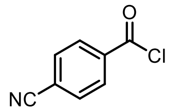 828 mg (5.00 mmol) of **2h**	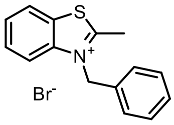 1.76 g (5.50 mmol) of **3a**	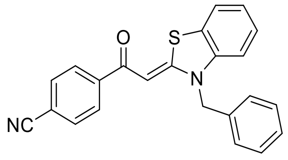 1.78 g (97%) of **1l**

entry	acid chloride **2**	benzothiazolium bromide **3**	**1** (yield)
13	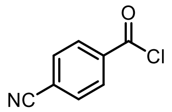<br>828 mg (5.00 mmol) of **2h**	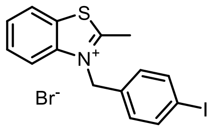<br>2.45 g (5.49 mmol) of **3b**	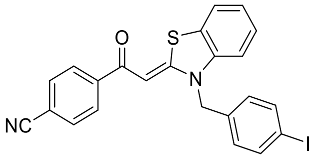<br>1.31 g (53%) of **1m**
14	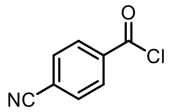<br>536 mg (3.24 mmol) of **2h**	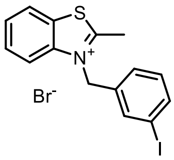<br>1.59 g (3.56 mmol) of **3c**	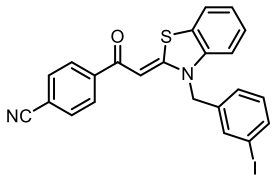<br>1.40 g (87%) of **1n**
15	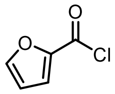<br>0.100 mL (1.01 mmol) of **2i**	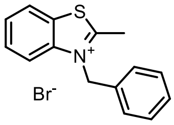<br>352 mg (1.10 mmol) of **3a**	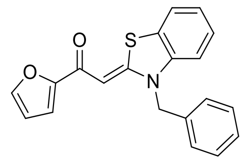<br>330 mg (98%) of **1o**
16	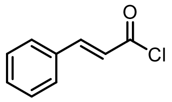<br>167 mg (1.00 mmol) of **2j**	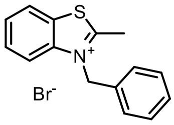<br>352 mg (1.10 mmol) of **3a**	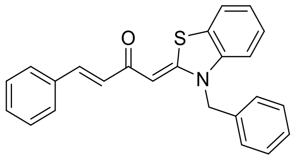<br>74 mg (20%) of **1p**

[a] 8 mL/mmol 1,4-dioxane were used.

2.2.2 Spectroscopic data

(*Z*)-2-(3-Benzylbenzo[*d*]thiazol-2(3*H*)-ylidene)-1-(4-methoxyphenyl)ethan-1-one (1a) [4]

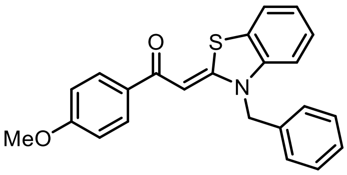

$C_{23}H_{19}NO_2S$ [373.47]

The synthesis was performed according to **GP II** to give 1.68 g (4.50 mmol, 90%) of the desired product **1a** as a yellow solid, Mp 218 °C (lit. 210 °C) [4]. R_f (*n*-hexane/acetone 3:1): 0.39.

^{1}H NMR (600 MHz, $CDCl_3$): δ 3.83 (s, 3 H), 5.32 (s, 2 H), 6.55 (s, 1 H), 6.87 - 6.91 (m, 2 H), 7.09 (d, J = 8.1 Hz, 1 H), 7.19 (td, ^3J = 7.5 Hz, ^4J = 1.0 Hz, 1 H), 7.21 - 7.25 (m, 2 H), 7.27 - 7.32 (m, 2 H), 7.33 - 7.38 (m, 2 H), 7.65 (dd, ^3J = 7.8 Hz, ^4J = 1.2 Hz, 1 H), 7.85 - 7.90 (m, 2 H).

^{13}C NMR (150 MHz, $CDCl_3$): δ 49.6 (CH_2), 55.5 (CH_3), 87.5 (CH), 110.1 (CH), 113.6 (CH), 122.6 (CH), 123.1 (CH), 126.4 (CH), 126.7 (CH), 127.5 (C_{quat}), 128.2 (CH), 129.2 (CH), 129.3 (CH), 132.4 (C_{quat}), 134.4 (C_{quat}), 140.1 (C_{quat}), 161.9 (C_{quat}), 162.0 (C_{quat}), 184.2 (C_{quat}).

MALDI-TOF (*m/z*): 374.1 ($C_{23}H_{19}NO_2S+H^+$).

(*Z*)-2-(3-(4-Iodobenzyl)benzo[*d*]thiazol-2(3*H*)-ylidene)-1-(4-methoxyphenyl)ethan-1-one (1b)

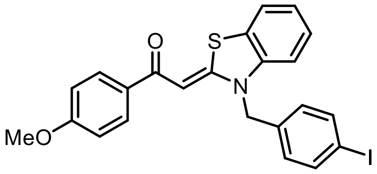

$C_{23}H_{18}INO_2S$ [499.37]

The synthesis was performed according to **GP II** to give 1.71 g (3.42 mmol, 68%) of the desired product **1b** as a yellow solid, Mp 196 °C. R_f (*n*-hexane/acetone 3:1): 0.50.

^{1}H NMR (600 MHz, $CDCl_3$): δ 3.84 (s, 3 H), 5.26 (s, 2 H), 6.50 (s, 1 H), 6.88 - 6.93 (m, 2 H), 6.95 - 6.99 (m, 2 H), 7.04 (d, ^3J = 8.1 Hz, 1 H), 7.17 - 7.23 (m, 1 H), 7.30 (ddd, ^3J = 8.4 Hz, ^3J = 7.4 Hz, ^4J = 1.2 Hz, 1 H), 7.63 - 7.69 (m, 3 H), 7.85 - 7.90 (m, 2 H).

^{13}C NMR (150 MHz, $CDCl_3$): δ 49.1 (CH_2), 55.5 (CH_3), 87.4 (CH), 93.7 (C_{quat}), 109.9 (CH), 113.7 (CH), 122.7 (CH), 123.3 (CH), 126.7 (CH), 127.5 (C_{quat}), 128.3 (CH), 129.2 (CH), 132.3 (C_{quat}), 134.1 (C_{quat}), 138.4 (CH), 139.8 (C_{quat}), 161.8 (C_{quat}), 162.1 (C_{quat}), 184.3 (C_{quat}).

MALDI-TOF (*m/z*): 500.1 ([$C_{23}H_{18}INO_2S$ + H]$^+$).

IR $\tilde{\nu}$ [cm^{-1}]: 3078 (w), 3063 (w), 3001 (w), 2932 (w), 2837 (w), 1597 (w), 1557 (s), 1530 (w), 1508 (w), 1470 (s), 1462 (s), 1454 (s), 1447 (s), 1414 (m), 1400 (s), 1393 (s), 1342 (m), 1337 (m), 1331 (m), 1306 (m), 1292 (m), 1250 (m), 1225 (s), 1194 (m), 1186 (m), 1167 (s), 1138 (m), 1119 (w), 1109 (m), 1088 (s), 1067 (m), 1063 (m), 1045 (m), 1024 (s), 1005 (s), 970 (w), 945 (w), 924 (w), 880 (s), 835 (s), 824 (m), 816 (m), 808 (m), 791 (m), 775 (w), 762 (s), 741 (s), 719 (s), 708 (s), 698 (s), 687 (m), 681 (m), 664 (m), 615 (s).

UV/Vis (C_2H_5OH): λ_{max} (ε) = 383 (33800).

Anal calcd for $C_{23}H_{18}INO_2S$ [499.4]: C 55.32, H 3.63, N 2.80, S 6.42; **Found:** C 55.20, H 3.67, N 2.75, S 6.58.

(*Z*)-2-(3-Benzylbenzo[*d*]thiazol-2(3*H*)-ylidene)-1-(*m*-tolyl)ethan-1-one (1c) [5]

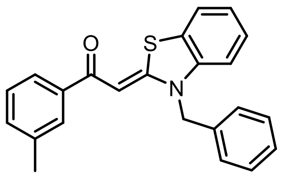

$C_{23}H_{19}NOS$ [357.47]

The synthesis was performed according to **GP II** to give 339 mg (0.948 mmol, 96%) of the desired product **1c** as a yellow solid, Mp 186 °C (lit. 162 °C) [5]. R_f (*n*-hexane/acetone 3:1): 0.49.

^{1}H NMR (600 MHz, $CDCl_3$): δ 2.36 (s, 3 H), 5.33 (s, 2 H), 6.57 (s, 1 H), 7.09 (d, ^3J = 8.1 Hz, 1 H), 7.17 - 7.25 (m, 5 H), 7.277 - 7.31 (m, 2 H), 7.317 - 7.36 (m, 2 H), 7.627 - 7.67 (m, 2 H), 7.70 (s, 1 H).

^{13}C NMR (150 MHz, $CDCl_3$): δ 21.6 (CH_3), 49.7 (CH_2), 88.0 (CH), 110.2 (CH), 122.7 (CH), 123.2 (CH), 124.4 (CH), 126.5 (CH), 126.7 (CH), 127.5 (C_{quat}), 128.1 (CH), 128.3 (CH), 129.3 (CH), 131.8 (CH), 134.3 (C_{quat}), 138.1 (C_{quat}., 139.7 (C_{quat}), 140.0 (C_{quat}), 162.3 (C_{quat}), 185.4 (C_{quat}).

MALDI-TOF (*m/z*): 358.2 ($C_{23}H_{19}NOS$+H$^+$).

(*Z*)-2-(3-Benzylbenzo[*d*]thiazol-2(3*H*)-ylidene)-1-(4-hexylphenyl)ethan-1-one (1d) [5]

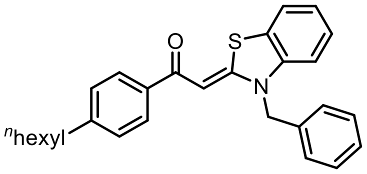

$C_{28}H_{29}INOS$ [427.61]

The synthesis was performed according to **GP II** to give 1.02 g (2.38 mmol, 95%) of the desired product **1d** as a yellow solid, Mp 102 °C (lit. 99 °C) [5]. R_f (*n*-hexane/acetone 3:1): 0.48.

^{1}H NMR (300 MHz, $CDCl_3$): δ 0.87 (t, ^3J = 6.8 Hz, 3 H), 1.25 - 1.36 (m, 6 H), 1.56 - 1.66 (m, 2 H), 2.62 (t, ^3J = 7.6 Hz, 2 H), 5.33 (s, 2 H), 6.59 (s, 1 H), 7.11 (dd, ^3J = 8.2 Hz, ^4J = 1.1 Hz, 1 H), 7.17 - 7.26 (m, 5 H), 7.28 - 7.39 (m, 4 H), 7.66 (dd, ^3J = 7.7 Hz, ^4J = 1.3 Hz, 1 H), 7.77 - 7.85 (m, 2 H).

^{13}C NMR (75 MHz, $CDCl_3$): δ 14.2 (CH_3), 22.7 (CH_2), 29.1 (CH_2), 31.3 (CH_2), 31.8 (CH_2), 36.0 (CH_2), 49.6 (CH_2), 87.8 (CH), 110.1 (CH), 122.6 (CH), 123.1 (CH), 126.4 (CH), 126.7 (CH), 127.4 (CH), 127.5 (C_{quat}), 128.2 (CH), 128.5 (CH), 129.3 (CH), 134.3 (C_{quat}), 137.2 (C_{quat}), 140.1 (C_{quat}), 146.4 (C_{quat}), 162.1 (C_{quat}), 185.0 (C_{quat}).

MALDI-TOF (*m/z*): 428.3 ($[C_{28}H_{29}NOS + H]^+$).

(*Z*)-1-(4-Hexylphenyl)-2-(3-(4-iodobenzyl)benzo[*d*]thiazol-2(3*H*)-ylidene)ethan-1-one (1e)

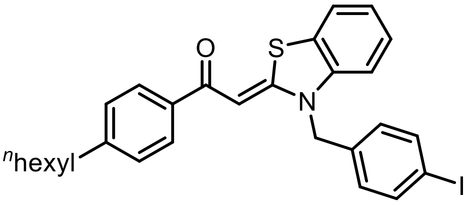

$C_{28}H_{28}INOS$ [553.50]

The synthesis was performed according to **GP II** to give 1.14 g (4.05 mmol, 81%) of the desired product **1e** as a yellow solid, Mp 175 °C. R_f (*n*-hexane/acetone 3:1): 0.40.

^{1}H NMR (300 MHz, $CDCl_3$): δ 0.77 - 0.98 (m, 3 H), 1.17 - 1.43 (m, 6 H), 1.48 - 1.70 (m, 2 H), 2.52 - 2.73 (m, 2 H), 5.27 (s, 2 H), 6.53 (s, 1 H), 6.91 - 7.13 (m, 3 H), 7.18 - 7.25 (m, 3 H), 7.31 (td, ^3J = 7.7 Hz, ^4J = 1.3 Hz, 1 H), 7.67 (dq, ^3J = 7.7 Hz, ^4J = 1.8 Hz, 3 H), 7.76 - 7.88 (m, 2 H).

^{13}C NMR (150 MHz, $CDCl_3$): δ 14.2 (CH_3), 22.7 (CH_2), 29.1 (CH_2), 31.3 (CH_2), 31.8 (CH_2), 36.0 (CH_2), 49.1 (CH_2), 87.7 (CH), 93.7 (C_{quat}), 109.9 (CH), 122.8 (CH), 123.3 (CH), 126.8 (CH),

127.4 (CH), 127.5 (C_{quat}), 128.3 (CH), 128.5 (CH), 134.0 (C_{quat}), 137.1 (C_{quat}), 138.4 (CH), 139.8 (C_{quat}), 146.6 (C_{quat}), 162.0 (C_{quat}), 185.1 (C_{quat}).

MALDI-TOF (*m/z*): 554.2 ($[C_{28}H_{28}INOS + H]^+$).

IR $\tilde{\nu}$ [cm^{-1}]: 2957 (w), 2924 (w), 2857 (w), 1593 (m), 1557 (m), 1510 (w), 1466 (s), 1449 (s), 1400 (m), 1377 (w), 1348 (w), 1329 (m), 1302 (w), 1260 (w), 1229 (m), 1190 (m), 1177 (m), 1157 (w), 1136 (w), 1117 (w), 1092 (w), 1069 (w), 1057 (w), 1040 (w), 1018 (w), 1003 (m), 966 (w), 953 (w), 918 (w), 880 (s), 827 (m), 783 (w), 850 (s), 714 (m), 708 (m), 656 (w), 637 (w).

UV/Vis (C_2H_5OH): λ_{max} (ε) = 381 (51500).

Anal calcd for $C_{28}H_{28}INOS$ [553.5]: C 60.76, H 5.10, N 2.53, S 5.79; **Found:** C 60.46, H 5.08, N 2.61, S 6.00.

(*Z*)-2-(3-Benzylbenzo[*d*]thiazol-2(3*H*)-ylidene)-1-phenylethan-1-one (1f) [4]

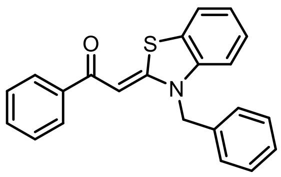

$C_{22}H_{17}NOS$ [343.44]

The synthesis was performed according to **GP II** to give 329 mg (0.958 mmol, 93%) of the desired product **1f** as a yellow solid. Mp 166 °C (lit. 161 °C) [4]. R_f (*n*-hexane/acetone 3:1): 0.28.

^{1}H NMR (600 MHz, $CDCl_3$): δ 5.35 (s, 2 H), 6.60 (s, 1 H), 7.13 (d, J^3 = 8.1 Hz, 1 H), 7.19 - 7.26 (m, 3 H), 7.28 - 7.46 (m, 7 H), 7.68 (dd, ^3J = 7.9 Hz, ^4J = 1.2 Hz, 1 H), 7.87 - 7.92 (m, 2 H).

^{13}C NMR (150 MHz, $CDCl_3$): δ 49.7 (CH_2), 87.8 (CH), 110.2 (CH), 122.7 (CH), 123.3 (CH), 126.4 (CH), 126.8 (CH), 127.3 (CH), 127.5 (C_{quat}), 128.3 (CH), 128.4 (CH), 129.4 (CH), 131.0 (CH), 134.3 (C_{quat}), 139.7 (C_{quat}), 140.0 (C_{quat}), 162.4 (C_{quat}), 185.1 (C_{quat}).

MALDI-TOF (*m/z*): 344.120 ($[C_{22}H_{17}NOS + H]^+$).

(*Z*)-2-(3-(4-Iodobenzyl)benzo[*d*]thiazol-2(3*H*)-ylidene)-1-phenylethan-1-one (1g) [5]

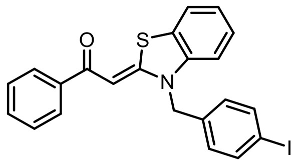

$C_{22}H_{16}INOS$ [469.34]

The synthesis was performed according to **GP II** to give 2.32 g (4.95 mmol, 99%) of the desired product **1g** as a yellow solid, Mp 175 °C (lit. 172 °C) [5]. R_f (*n*-hexane/acetone 3:1): 0.39.

^{1}H NMR (300 MHz, $CDCl_3$): δ 5.11 (s, 2 H), 6.38 (s, 1 H), 6.75 - 6.85 (m, 2 H), 6.86 - 6.95 (m, 1 H), 7.05 (td, ^4J = 7.5 Hz, ^4J = 1.1 Hz, 1 H), 7.12 - 7.18 (m, 1 H), 7.19 - 7.31 (m, 3 H), 7.45 - 7.56 (m, 3 H), 7.66 - 7.79 (m, 2 H).

^{13}C NMR (150 MHz, $CDCl_3$): δ 49.1 (CH_2), 87.8 (CH), 93.7 (C_{quat}), 110.0 (CH), 122.8 (CH), 123.4 (CH), 126.9 (CH), 127.3 (CH), 127.4 (C_{quat}), 128.3 (CH), 128.5 (C_{quat}), 131.1 (CH), 133.9 (C_{quat}), 138.4 (CH), 139.6 (C_{quat}), 139.7 (C_{quat}), 162.3 (C_{quat}), 185.2 (C_{quat}).

MALDI-TOF (*m/z*): 470.1 ([$C_{22}H_{16}INOS$ + H]$^+$).

(*Z*)-2-(3-Benzyl-5,6-dimethylbenzo[*d*]thiazol-2(3*H*)-ylidene)-1-phenylethan-1-one (1h) [5]

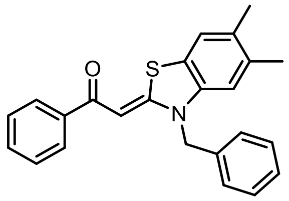

$C_{24}H_{21}NOS$ [371.50]

The synthesis was performed according to **GP II** to give 353 mg (0.950 mmol, 92%) of the desired product **1h** as a yellow solid, Mp 278 °C (lit. 282 °C) [5]. R_f (*n*-hexane/acetone 3:1): 0.39.

^{1}H NMR (600 MHz, $CDCl_3$): δ 2.30 (s, 3 H), 2.32 (s, 3 H), 5.32 (s, 2 H), 6.53 (s, 1 H), 6.92 (s, 1 H), 7.22 (d, ^3J = 7.5 Hz, 2 H), 7.28 - 7.33 (m, 1 H), 7.33 - 7.45 (m, 6 H), 7.88 (d, ^3J = 7.0, Hz, 2 H).

^{13}C NMR (150 MHz, $CDCl_3$): δ 19.7 (CH_3), 20.5 (CH_3), 49.6 (CH_2), 87.5 (CH), 111.2 (CH), 123.2 (CH), 124.6 (C_{quat}), 126.4 (CH), 127.3 (CH), 128.2 (CH), 128.3 (CH), 129.3 (CH), 130.8

(CH), 132.3 (C_{quat}), 134.5 (C_{quat}), 135.8 (C_{quat}), 138.4 (C_{quat}), 139.9 (C_{quat}), 162.6 (C_{quat}), 184.7 (C_{quat}).

MALDI-TOF (*m/z*): 372.2 ($C_{24}H_{21}NOS+H^+$).

(*Z*)-2-(3-Benzylbenzo[*d*]thiazol-2(3*H*)-ylidene)-1-(4-fluorophenyl)ethan-1-one (1i) [4]

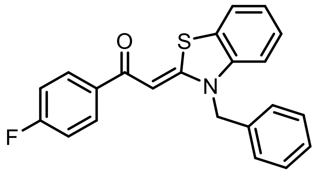

$C_{22}H_{16}FNOS$ [361.43]

The synthesis was performed according to **GP II** to give 1.78 g (4.93 mmol, 99%) of the desired product **1i** as a yellow solid, Mp 158 °C (lit. 155 °C) [4]. R_f (*n*-hexane/acetone 3:1): 0.49.

^{1}H NMR (300 MHz, $CDCl_3$): δ 5.27 (s, 2 H), 6.45 (s, 1 H), 6.92 - 7.09 (m, 3 H), 7.11 - 7.20 (m, 3 H), 7.20 - 7.34 (m, 4 H), 7.55 - 7.65 (m, 1 H), 7.74 - 7.90 (m, 2 H).

^{13}C NMR (150 MHz, $CDCl_3$): δ 49.7 (CH_2), 87.4 (CH), 110.3 (CH), 115.3 (d, ^2J = 21.6, Hz, CH), 122.7 (CH), 123.3 (CH), 126.4 (CH), 126.8 (CH), 127.4 (C_{quat}), 128.3 (CH), 129.4 (CH), 129.5 (d, ^3J = 8.8 Hz, CH), 134.2 (C_{quat}), 135.9 (d, ^4J = 3.0 Hz, C_{quat}), 140.0 (C_{quat}), 162.6 (C_{quat}), 164.6 (d, ^1J = 251.6, Hz, C_{quat}), 183.6 (C_{quat}).

MALDI-TOF (*m/z*): 362.1 ($C_{22}H_{16}FNOS+H^+$).

(*Z*)-2-(3-Benzylbenzo[*d*]thiazol-2(3*H*)-ylidene)-1-(2,4-dichlorophenyl)ethan-1-one (1j) [5]

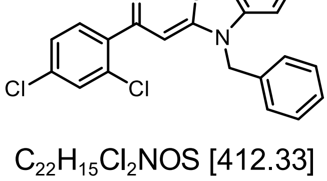

$C_{22}H_{15}Cl_2NOS$ [412.33]

The synthesis was performed according to **GP II** to give 403 mg (0.977 mmol, 98%) of the desired product **1j** as a yellow solid, Mp 166 °C (lit. 167 °C) [5]. R_f (*n*-hexane/acetone 3:1): 0.35.

^{1}H NMR (600 MHz, $CDCl_3$): δ (s, 2 H), 6.28 (s, 1 H), 7.08 - 7.13 (m, 3 H), 7.15 - 7.20 (m, 2 H), 7.20 - 7.30 (m, 5 H), 7.46 (dd, ^3J = 8.3 Hz, 1 H), 7.62 (d, ^3J = 7.8 Hz, 1 H).

^{13}**C NMR (150 MHz, CDCl$_3$):** δ 49.7 (CH_2), 92.1 (CH), 110.5 (CH), 122.8 (CH), 123.6 (CH), 126.5 (CH), 127.0 (CH), 127.2 (CH), 127.2 (C_{quat}), 128.3 (CH), 129.3 (CH), 130.0 (CH), 131.2 (CH), 132.1 (C_{quat}), 134.1 (C_{quat}), 135.6 (C_{quat}), 139.2 (C_{quat}), 139.9 (C_{quat}), 162.2 (C_{quat}), 183.9 (C_{quat}).

MALDI-TOF (*m/z*): 412.1 ($C_{22}H_{15}Cl_2NOS+H^+$).

(*Z*)-2-(3-Benzylbenzo[*d*]thiazol-2(3*H*)-ylidene)-1-(5-chloro-2-fluorophenyl)ethan-1-one (1k) [5]

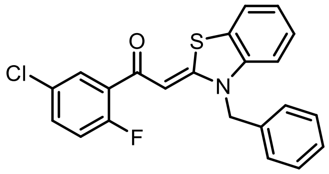

$C_{22}H_{15}ClFNOS$ [395.88]

The synthesis was performed according to **GP II** to give 387 mg (0.978 mmol, 99%) of the desired product **1k** as a yellow solid, Mp 173 °C (lit. 177 °C) [5]. R_f (*n*-hexane/acetone 3:1): 0.50.

^{1}**H NMR (600 MHz, CDCl$_3$):** δ 5.28 (s, 2 H), 6.55 (s, 1 H) 6.89 - 6.96 (m, 1 H), 7.11 - 7.20 (m, 4 H), 7.21 - 7.32 (m, 5 H), 7.63 (d, ^3J = 7.8 Hz, 1 H), 7.88 - 7.94 (m, 1 H).

^{13}**C NMR (150 MHz, CDCl$_3$):** δ 49.8 (CH_2), 92.1 (d, ^3J = 11.6 Hz, CH), 110.6 (CH), 117.7 (d, ^2J = 26.4 Hz, CH), 122.8 (CH), 123.6 (CH), 126.6 (CH), 127.1 (CH), 127.5 (C_{quat}), 128.4 (CH), 129.1 (d, ^2J = 14.6 Hz, C_{quat}), 129.3 (CH), 129.8 (d, ^4J = 3.1 Hz, C_{quat}), 131.8 (d, ^3J = 9.0 Hz, CH), 134.1 (C_{quat}), 140.0 (C_{quat}), 158.4 (C_{quat}), 161.5 (d, ^1J = 252.5 Hz, C_{quat}), 179.0 (C_{quat}), 179.0 (C_{quat}).

MALDI-TOF (*m/z*): 396.1 ($C_{22}H_{15}ClFNOS+H^+$).

(*Z*)-4-(2-(3-Benzylbenzo[*d*]thiazol-2(3*H*)-ylidene)acetyl)benzonitrile (1l) [4]

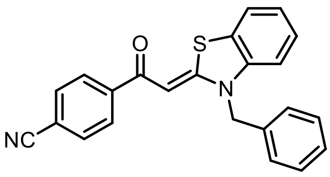

$C_{23}H_{16}N_2OS$ [368.45]

The synthesis was performed according to **GP II** to give 1.78 g (4.83 mmol, 97%) of the desired product **1l** as a yellow solid, Mp 203 °C (lit. 198 °C) [4]. R_f (*n*-hexane/acetone 3:1): 0.40.

^{1}H NMR (600 MHz, $CDCl_3$): δ 5.39 (s, 2 H), 6.55 (s, 1 H), 7.16 - 7.42 (m, 8 H), 7.65 - 7.77 (m, 3 H), 7.91 - 8.02 (m, 2 H).

^{13}C NMR (150 MHz, $CDCl_3$): δ 49.8 (CH_2), 87.8 (CH), 110.7 (CH), 114.0 (C_{quat}), 118.7 (C_{quat}), 122.9 (CH), 123.8 (CH), 126.3 (CH), 127.2 (CH), 127.3 (C_{quat}), 127.8 (CH), 128.5 (CH), 129.5 (CH), 132.3 (CH), 133.9 (C_{quat}), 139.8 (C_{quat}), 143.4 (C_{quat}), 163.5 (C_{quat}), 182.6 (C_{quat}).

MALDI-TOF (*m/z*): 369.1 ($C_{23}H_{16}N_2OS+H^+$).

(*Z*)-4-(2-(3-(4-Iodobenzyl)benzo[*d*]thiazol-2(3*H*)-ylidene)acetyl)benzonitrile (1m) [5]

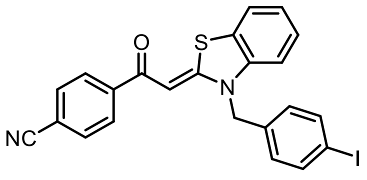

$C_{23}H_{15}IN_2OS$ [493.99]

The synthesis was performed according to **GP II** to give 1.31 g (2.65 mmol, 53%) of the desired product **1m** as a yellow solid, Mp 254 °C (lit. 235 °C) [5]. R_f (*n*-hexane/acetone 3:1): 0.30.

^{1}H NMR (300 MHz, $CDCl_3$): δ 5.33 (s, 2 H), 6.50 (s, 1 H), 6.88 - 7.01 (m, 2 H), 7.09 - 7.17 (m, 1 H), 7.30 (dd, ^3J = 7.6 Hz, ^4J = 1.1 Hz, 1 H), 7.34 - 7.41 (m, 1 H), 7.65 - 7.77 (m, 5 H), 7.89 - 8.03 (m, 2 H).

^{13}C NMR (150 MHz, $CDCl_3$): δ 49.3 (CH_2), 87.7 (CH), 93.9 (C_{quat}), 110.5 (CH), 114.2 (C_{quat}), 118.7 (C_{quat}), 123.0 (CH), 124.0 (CH), 127.2 (CH), 127.8 (CH), 128.2 (CH), 132.4 (CH), 133.5 (C_{quat}), 138.6 (CH), 139.6 (C_{quat}), 143.3 (C_{quat}), 163.4 (C_{quat}), 182.7 (C_{quat}).

MALDI-TOF (*m/z*): 495.1 ($C_{23}H_{15}IN_2OS+H^+$).

(*Z*)-4-(2-(3-(3-Iodobenzyl)benzo[*d*]thiazol-2(3*H*)-ylidene)acetyl)benzonitrile (1n)

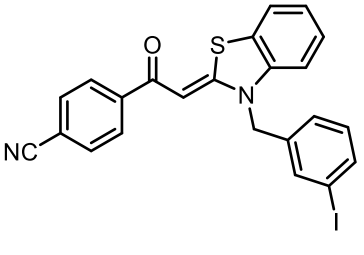

$C_{23}H_{15}IN_2OS$ [494.35]

The synthesis was performed according to **GP II** to give 1.40 g (2.83 mmol, 87%) of the desired product **1n** as a yellow solid. Mp 240 °C. R_f (*n*-hexane/acetone 3:1): 0.42.

^{1}H NMR (300 MHz, CDCl$_3$): δ 5.33 (s, 2 H), 6.51 (s, 1 H), 7.04 - 7.19 (m, 3 H), 7.29 (td, ^3J = 7.5 Hz, ^4J = 1.1 Hz, 1 H), 7.35 - 7.44 (m, 1 H), 7.60 - 7.78 (m, 5 H), 7.91 - 8.02 (m, 2 H).

^{13}C NMR (150 MHz, CDCl$_3$): δ 49.0 (CH_2), 87.8 (CH), 95.2 (C_{quat}), 110.5 (CH), 114.2 (C_{quat}), 118.7 (C_{quat}), 123.0 (CH), 124.0 (CH), 125.4 (CH), 127.2 (C_{quat}), 127.3 (CH), 127.8 (CH), 131.2 (CH), 132.4 (CH), 135.4 (CH), 136.2 (C_{quat}), 137.7 (CH), 139.6 (C_{quat}), 143.3 (C_{quat}), 163.4 (C_{quat}), 182.8 (C_{quat}).

MALDI-TOF (*m/z*): 495.0 ([$C_{23}H_{15}IN_2OS$ + H]$^+$).

IR $\tilde{\nu}$ [cm^{-1}]: 3096 (w), 3059 (w), 2224 (w), 1904 (w), 1593 (m), 1555 (w), 1505 (m), 1470 (s), 1449 (s), 1435 (m), 1408 (m), 1396 (m), 1369 (w), 1329 (m), 1310 (w), 1296 (w), 1271 (m), 1263 (w), 1231 (m), 1196 (m), 1173 (m), 1157 (w), 1132 (w), 1109 (w), 1093 (m), 1070 (w), 1044 (m), 1013 (s), 995 (w), 972 (w), 955 (w), 935 (w), 881 (m), 849 (m), 816 (w), 793 (m), 781 (w), 762 (s), 739 (s), 722 (s), 702 (m), 681 (s), 644 (m).

UV/Vis (C_2H_5OH): λ_{max} (ε) = 397 (8100).

Anal calcd for $C_{23}H_{15}IN_2OS$ [494.3]: C 55.88, H 3.06, N 5.67, S 6.49; **Found:** C 55.86, H 3.12, N 5.64, S 6.79.

(*Z*)-2-(3-Benzylbenzo[*d*]thiazol-2(3*H*)-ylidene)-1-(furan-2-yl)ethan-1-one (1o) [4]

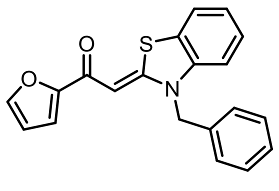

$C_{20}H_{15}NO_2S$ [333.41]

The synthesis was performed according to **GP II** to give 330 mg (0.990 mmol, 98%) of the desired product **1o** as a yellow solid, Mp 186 °C (lit. 201 °C) [4]. R_f (*n*-hexane/acetone 3:1): 0.32.

^{1}H NMR (600 MHz, CDCl$_3$): δ 5.34 (s, 2 H), 6.47 (s, 1 H), 6.57 (s, 1 H), 7.06 - 7.13 (m, 2 H), 7.18 - 7.25 (m, 3 H), 7.28 - 7.38 (m, 4 H), 7.44 (s, 1 H), 7.66 (d, ^3J = 7.7 Hz, 1 H).

^{13}**C NMR (150 MHz, CDCl$_3$):** δ 49.6 (CH_2), 87.4 (CH), 110.4 (CH), 112.2 (CH), 113.0 (CH), 122.7 (CH), 123.3 (CH), 126.5 (CH), 126.8 (CH), 127.6 (C_{quat}), 128.2 (CH), 129.3 (CH), 134.3 (C_{quat}), 139.9 (C_{quat}), 143.9 (CH), 154.6 (C_{quat}), 162.1 (C_{quat}), 174.9 (C_{quat}).

MALDI-TOF (*m/z*): 334.1 ($C_{20}H_{15}NO_2S+H^+$).

(1*Z*,3*E*)-1-(3-Benzylbenzo[*d*]thiazol-2(3*H*)-ylidene)-4-phenylbut-3-en-2-one (1p) [5]

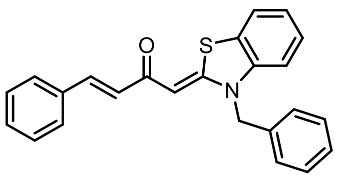

$C_{24}H_{19}NOS$ [369.48]

The synthesis was performed according to **GP II** to give 74 mg (0.20 mmol, 20%) of the desired product **1p** as a yellow solid, Mp 225 °C (lit. 77 °C) [5]. R_f (*n*-hexane/acetone 3:1): 0.30.

^{1}**H NMR (600 MHz, CDCl$_3$):** δ 5.32 (s, 2 H), 6.09 (s, 1 H), 6.81 (d, ^3J = 15.8 Hz, 1 H), 7.11 (d, ^3J = 8.1 Hz, 1 H), 7.19 - 7.24 (m, 3 H), 7.29 - 7.38 (m, 7 H), 7.52 - 7.57 (m, 2 H), 7.63 (d, ^3J = 15.7 Hz, 1 H), 7.68 (d, ^3J = 7.7 Hz, 1 H).

^{13}**C NMR (150 MHz, CDCl$_3$):** δ 49.6 (CH_2), 92.4 (CH), 110.3 (CH), 122.7 (CH), 123.3 (CH), 126.4 (CH), 126.8 (CH), 127.8 (C_{quat}), 127.9 (CH), 128.0 (CH), 128.3 (CH), 128.8 (CH), 129.3 (CH), 129.4 (CH), 134.2 (C_{quat}), 136.0 (C_{quat}), 138.6 (CH), 140.0 (C_{quat}), 161.8 (C_{quat}), 183.0 (C_{quat}).

MALDI-TOF (*m/z*): 370.2 ($C_{24}H_{19}NOS+H^+$).

3 NMR spectra

3.1 NMR spectra of benzothiazolium bromide 3c

^{1}H NMR spectrum of 3-(3-iodobenzyl)-2-methylbenzo[*d*]thiazol-3-ium bromide (3c) (DMSO-d$_6$, 300 MHz, 298 K)

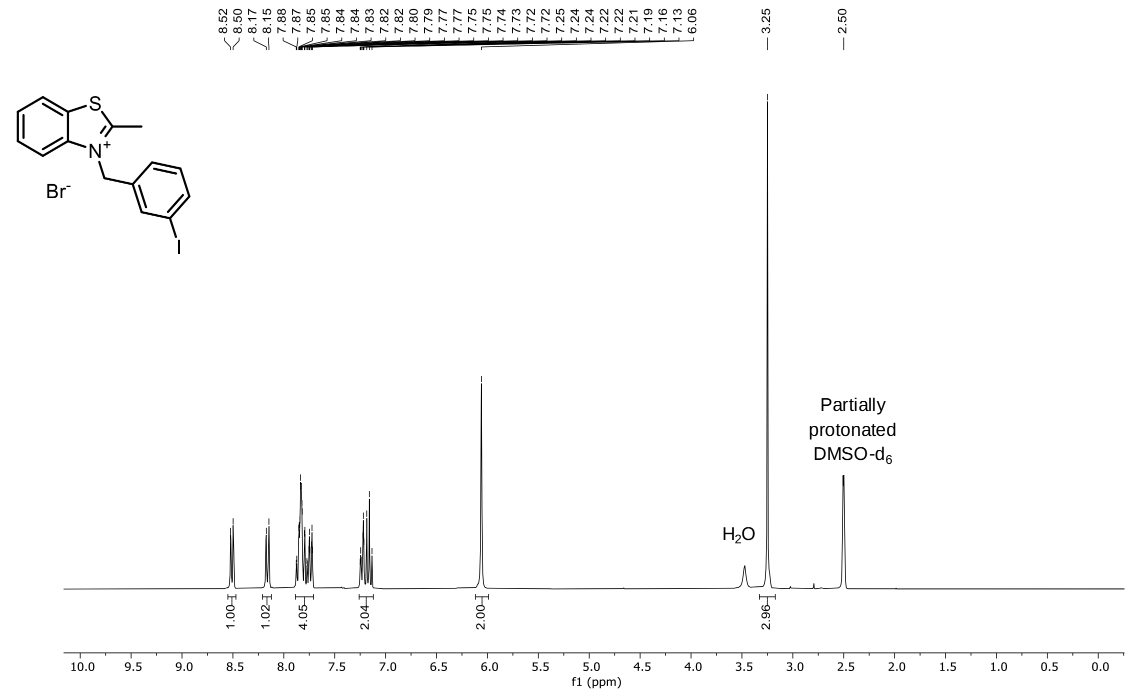

^{13}C NMR spectrum of 3-(3-iodobenzyl)-2-methylbenzo[*d*]thiazol-3-ium bromide (3c) (DMSO-d$_6$, 75 MHz, 298 K)

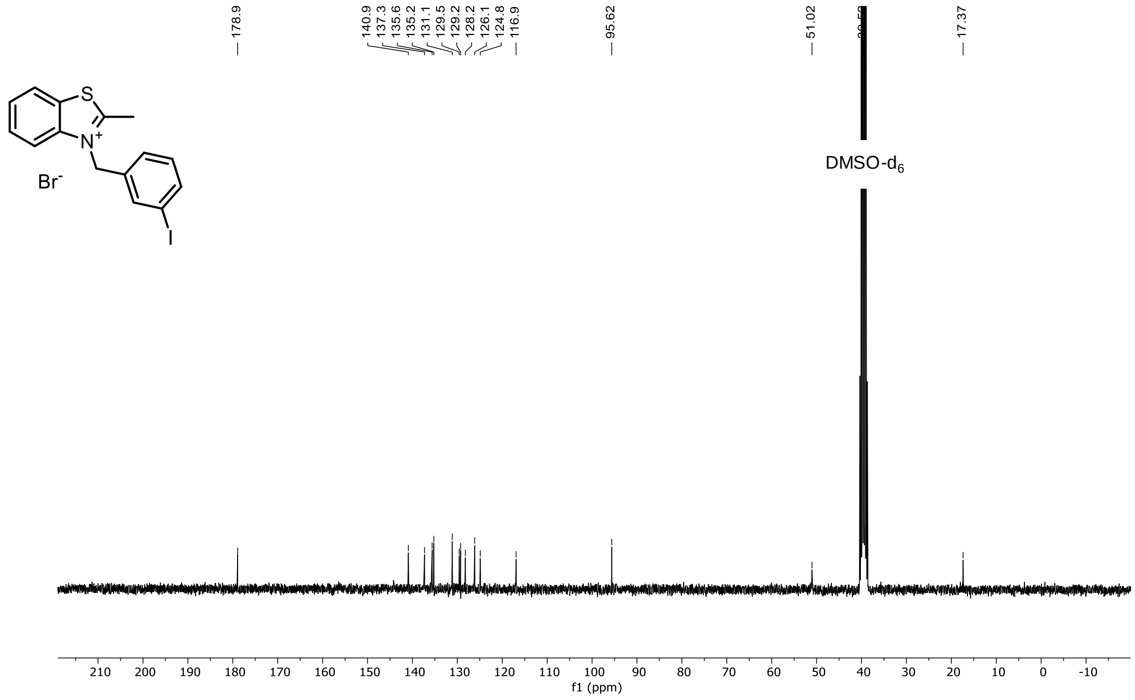

3.2 NMR spectra of aroyl-*S*,*N*-ketene acetals 1

^{1}H NMR spectrum of (*Z*)-2-(3-(4-iodobenzyl)benzo[*d*]thiazol-2(3*H*)-ylidene)-1-(4-methoxyphenyl)ethan-1-one (1b) ($CDCl_3$, 600 MHz, 298 K)

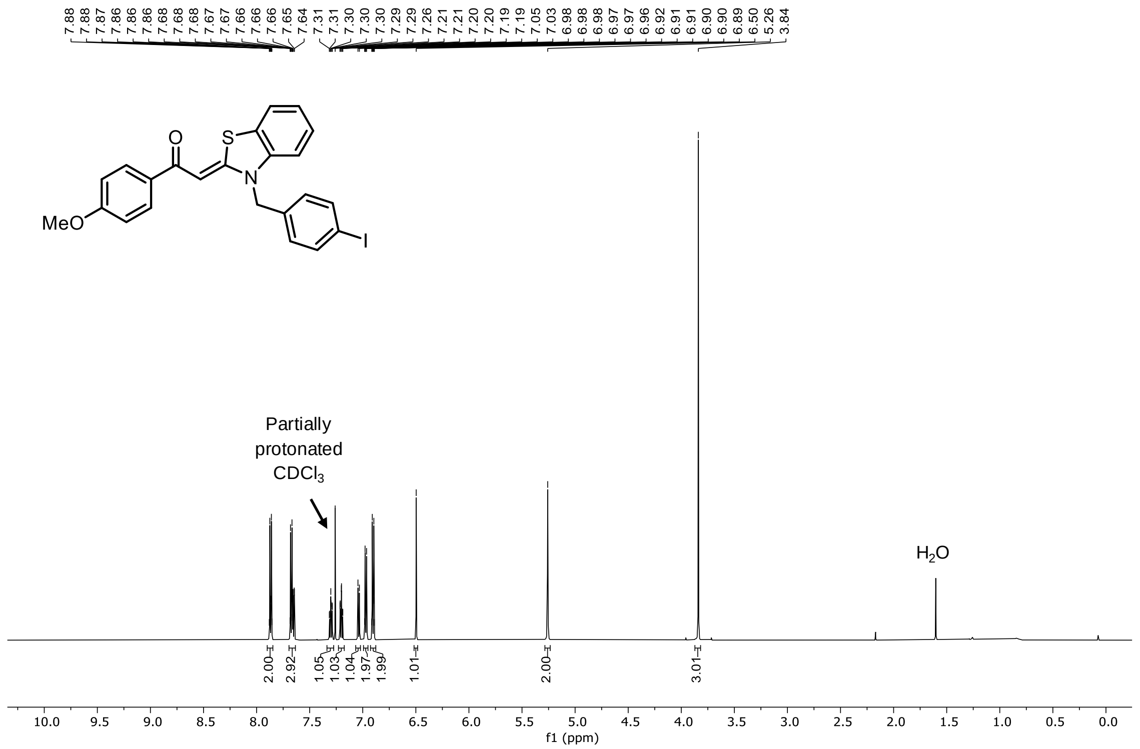

^{13}C NMR spectrum of (*Z*)-2-(3-(4-iodobenzyl)benzo[*d*]thiazol-2(3*H*)-ylidene)-1-(4-methoxyphenyl)ethan-1-one (1b) ($CDCl_3$, 150 MHz, 298 K)

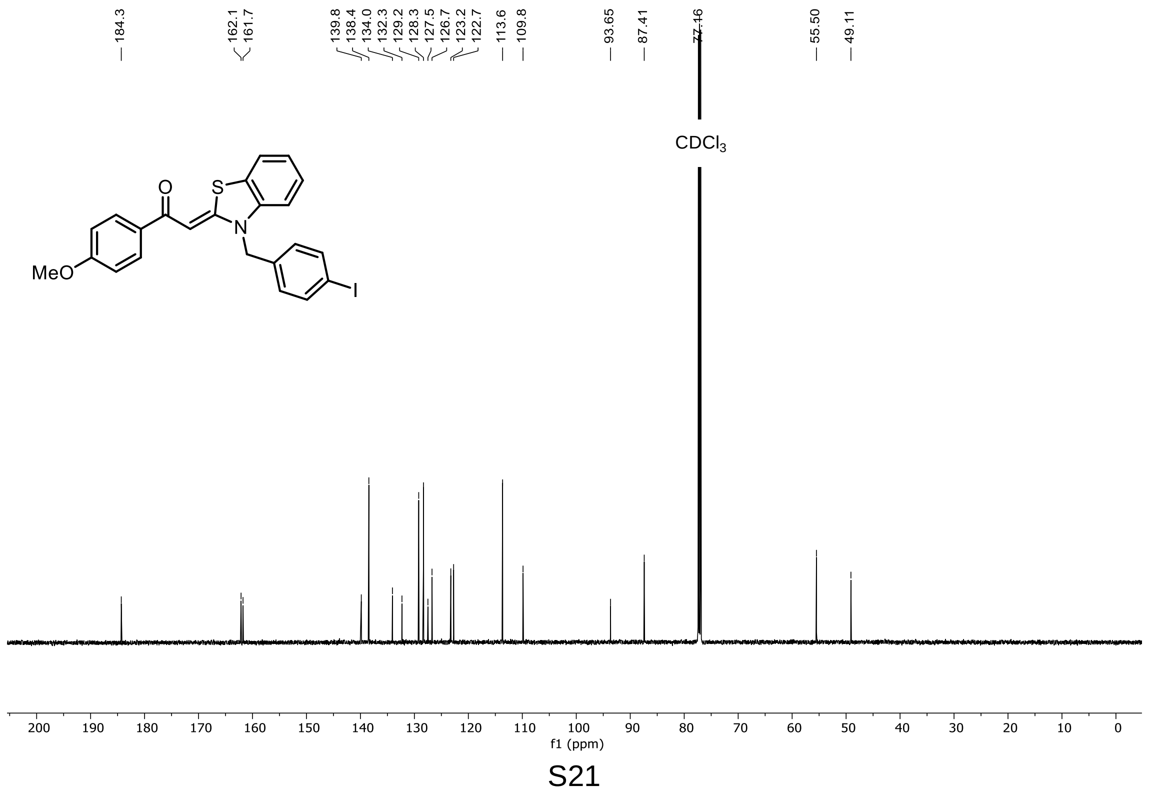

^{1}H NMR spectrum of (*Z*)-1-(4-hexylphenyl)-2-(3-(4-iodobenzyl)benzo[*d*]thiazol-2(3*H*)-ylidene)ethan-1-one (1e) ($CDCl_3$, 300 MHz, 298 K)

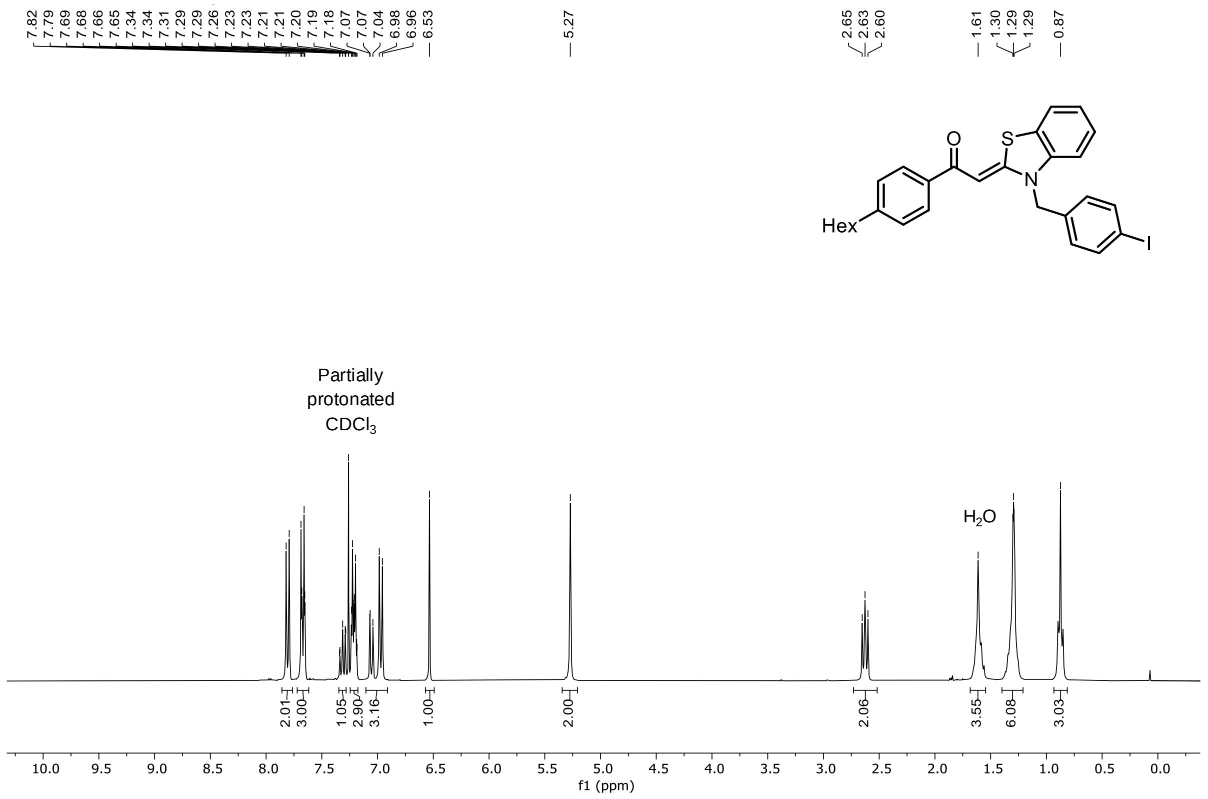

^{13}C NMR spectrum of (*Z*)-1-(4-hexylphenyl)-2-(3-(4-iodobenzyl)benzo[*d*]thiazol-2(3*H*)-ylidene)ethan-1-one (1e) ($CDCl_3$, 150 MHz, 298 K)

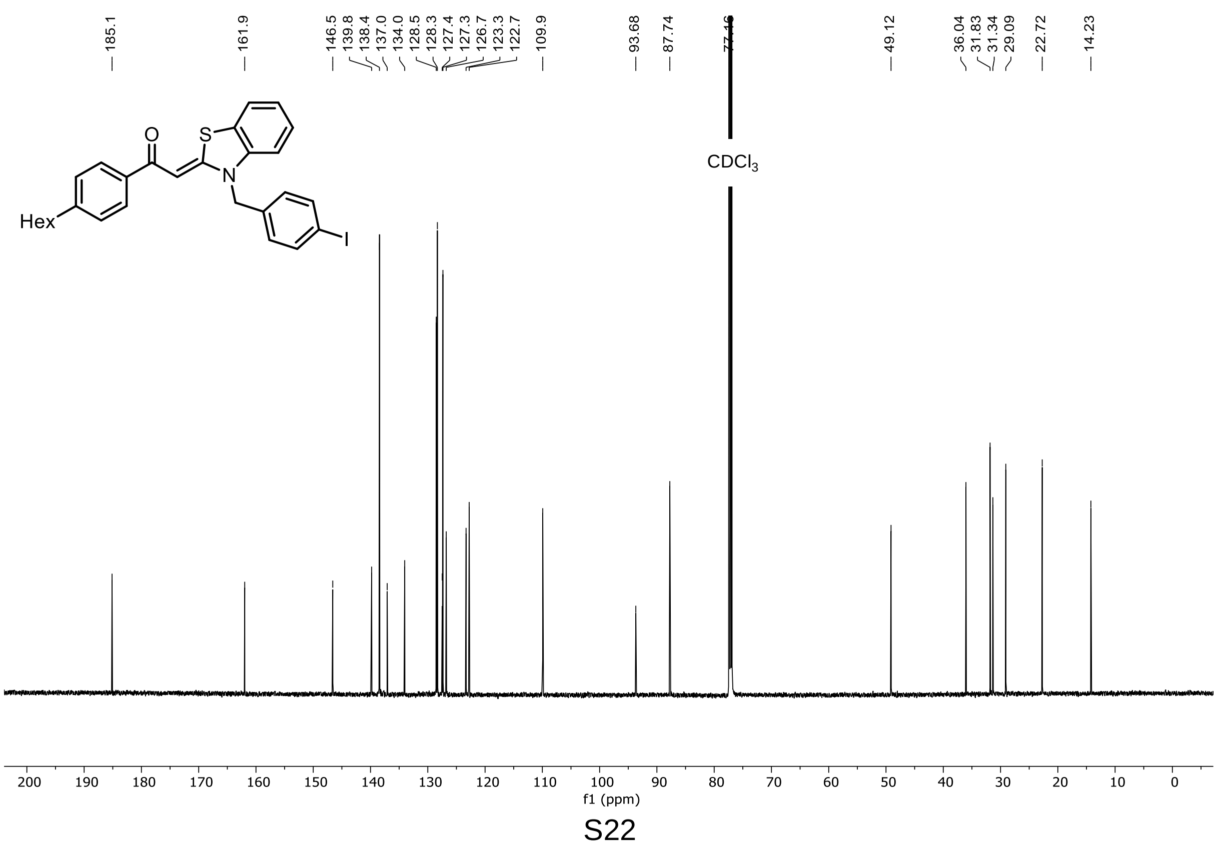

^{1}H NMR spectrum of (*Z*)-2-(3-(3-iodobenzyl)benzo[*d*]thiazol-2(3*H*)-ylidene)-1-(4-isocyanophenyl)ethan-1-one (1n) ($CDCl_3$, 300 MHz, 298 K)

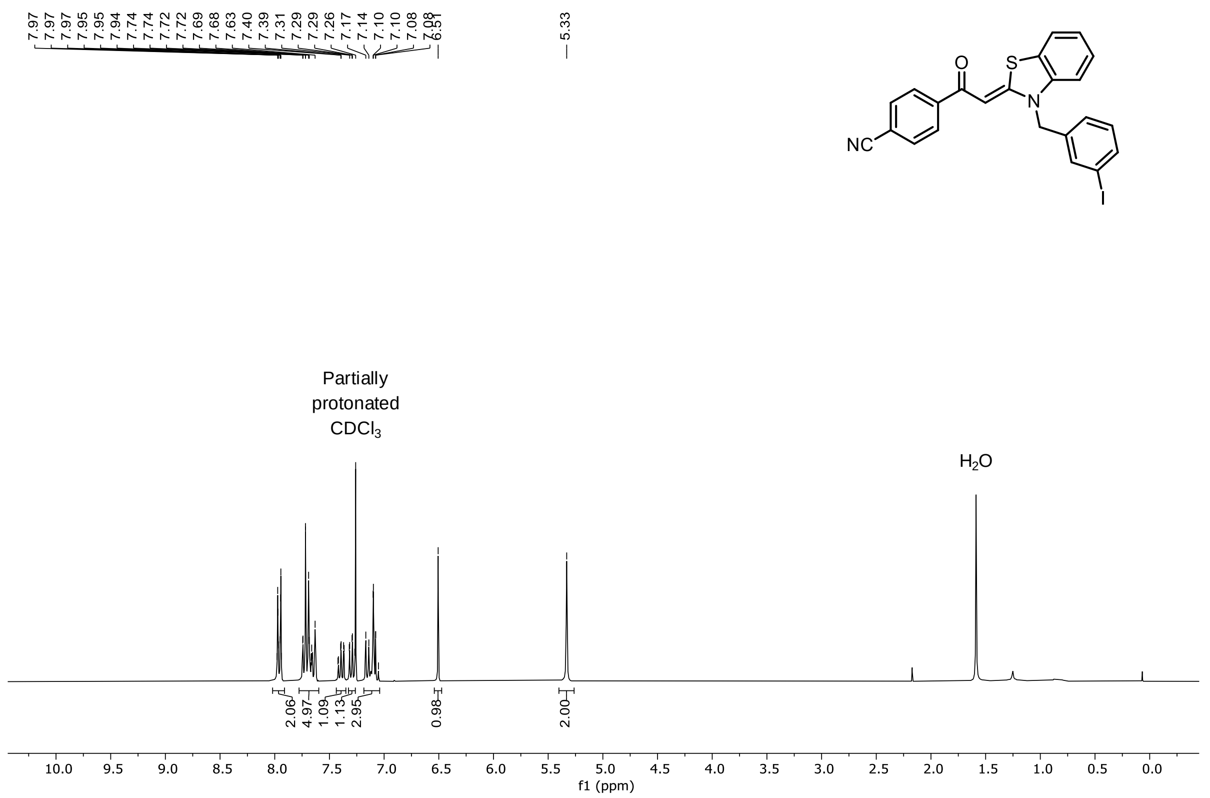

^{13}C NMR spectrum of (*Z*)-2-(3-(3-iodobenzyl)benzo[*d*]thiazol-2(3*H*)-ylidene)-1-(4-isocyanophenyl)ethan-1-one (1n) ($CDCl_3$, 150 MHz, 298 K)

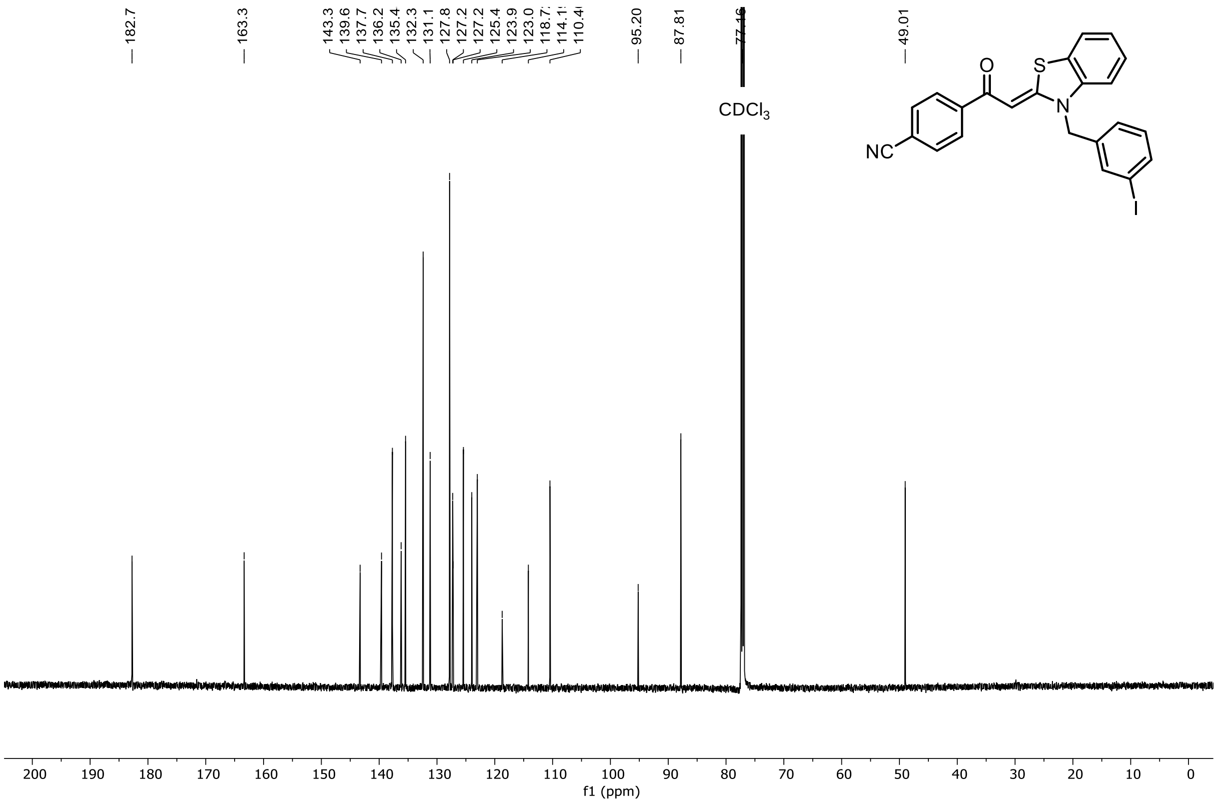

4 Literature

[1] Kuhnert-Brandstatter, M. *Sci. Pharm.* **1966**, *34*, 147-166.

[2] Hesse M.; Meier, H.; Zeeh, B. *Spektroskopische Methoden in der organischen Chemie*, Georg Thieme Verlag: Stuttgart, **2005**.

[3] Yen, S. K.; Koh, L. L.; Hahn F. E.; Huynh, H. V.; Hor, T. A. O*rganometallics* **2006**, *25*, 5105-5112. DOI: 10.1021/om060510n

[4] Biesen, L.; Nirmalananthan-Budau, N.; Hoffmann, K.; Resch-Genger, U.; Müller, T.J.J. *Angew. Chem.* **2020**, *132*, 10123-10127; *Angew. Chem. Int. Ed*. **2020**, *59*, 10037-10041. DOI: 10.1002/anie.201916396

[5] Biesen, L.; Woschko, D.; Janiak, C.; Müller, T. J. J. *Chem. Eur. J.* **2022**, *28*, e202202579. DOI: doi.org/10.1002/chem.202202579
